# Assessment of eHealth literacy among cardiovascular disease patients and analysis of influencing factors

**DOI:** 10.3389/fpubh.2025.1587163

**Published:** 2025-05-02

**Authors:** Wei Wang, Mengfan Jiao, Xiaojing Zhao, Chunxu Chen, Wenhui Jiang, Xiaoman Zhang

**Affiliations:** ^1^Department of Cardiology, Shandong Provincial Hospital Affiliated to Shandong First Medical University, Jinan, China; ^2^Department of Nursing, Shandong First Medical University, Taian, China; ^3^School of Nursing, Health Science Center, Xian Jiaotong University, Xi'an, China

**Keywords:** cardiovascular disease, health literacy, eHealth literacy, health education, influencing factors

## Abstract

**Objective:**

This study aimed to comprehensively evaluate the eHealth literacy of patients with cardiovascular diseases and uncover the associated influencing factors. The findings are intended to lay a solid foundation for formulating targeted strategies to enhance the health literacy of this patient population.

**Methods:**

Between October 2023 and June 2024, a purposive sampling approach was employed to recruit patients with cardiovascular diseases visiting the cardiology department of a tertiary hospital in Shandong Province. The eHealth Literacy Scale (eHEALS) was utilized to assess the eHealth literacy levels of the participants. Based on the assessment results, the subjects were classified into qualified and unqualified groups. Subsequently, logistic regression analysis was conducted to identify the influencing factors underlying eHealth literacy.

**Results:**

The eHealth literacy score among cardiovascular disease patients was 20.46 ± 9.54, with a passing rate of 38.6%. The overall mean score across all items was 2.5 ± 1.19. Specifically, for the sub-domains of internet health information service capabilities and application abilities, evaluation capabilities of internet health information and services, and decision - making capabilities of internet health information and services, the mean scores were 2.49 ± 1.18, 2.67 ± 1.32, and 2.66 ± 1.35, respectively. Findings from binary logistic regression analysis suggest that education level, sleep quality, residing in close proximity to a medical institution (distance < 5 km), prior utilization of medical information websites or search engines, as well as the interaction between proactive health awareness and utilization of medical information websites or search engines, were all influencing factors for the qualification of e - health literacy (*p* < 0.05). These results underscore the complex interplay of multiple factors in determining patients’ eHealth literacy levels, which has important implications for the design and implementation of effective health information dissemination and patient education strategies in the digital age.

**Conclusion:**

Our findings reveal that the eHealth literacy among cardiovascular disease (CVD) patients remains at a relatively low level. This situation underscores the urgent need for interventions aimed at enhancing patients’ proactive health awareness and delivering targeted eHealth training programs. Specifically, such initiatives should be designed to enable patients to accurately access, comprehensively understand, critically evaluate, and effectively apply health information in the digital realm. By doing so, we can empower CVD patients to better manage their health in the context of the digital age, ultimately leading to an improvement in their eHealth literacy levels. These efforts are not only crucial for individual patient care but also have broader implications for optimizing health outcomes at a population level.

## 1 Introduction

Chronic non-communicable diseases (NCDs), encompassing cardiovascular and cerebrovascular diseases, respiratory diseases, diabetes, malignant tumors, and mental health disorders, are a global health challenge of increasing significance (1). As per the “World Health Statistics 2021” report, chronic diseases account for 71.93% of all deaths, topping the list of mortality causes (2).

In China, among the older adult population aged 70 and above, cardiovascular and cerebrovascular diseases stand out as the leading contributor to disability-adjusted life years (39.11%), as indicated by relevant prevalence statistics (3). Cardiovascular diseases are characterized by an insidious onset, a protracted course, and a high recurrence rate, resulting in substantial disability and mortality. This not only places a heavy psychological burden on patients but also exacts a significant economic toll.

Given these circumstances, it is of utmost importance to implement appropriate strategies. These strategies should aim to assist chronic disease patients in accessing disease related information, effectively managing their conditions, and making well - informed health decisions. By doing so, we can potentially decelerate the progression of chronic diseases, thus improving patients’ quality of life and alleviating the burden on healthcare systems.

In the era of seamless integration between the internet and the healthcare domain, intelligent and digital technologies have emerged as pivotal pillars in chronic disease prevention (4). A plethora of information platforms now offer eHealth resources tailored for patients grappling with chronic cardiovascular diseases. The capacity of patients to actively seek, critically evaluate, comprehensively understand, and effectively apply these resources is encapsulated within the concept of eHealth literacy (5).

Accumulating evidence has firmly established a positive association between eHealth literacy and health - promoting behaviors (6–8). Given this connection, accurately gauging patients’ eHealth literacy levels and ensuring the targeted dissemination of eHealth resources assume paramount importance.

In recent years, research endeavors into eHealth literacy in the context of chronic diseases have witnessed a steady upsurge, covering a wide spectrum of themes (9). Nevertheless, the body of literature specifically dedicated to exploring eHealth literacy in cardiovascular diseases and other distinct chronic conditions remains relatively scarce.

Against this backdrop, the present study endeavors to employ a survey-based approach to evaluate the eHealth literacy levels of cardiovascular disease patients. Through this investigation, we aim to dissect the influencing factors and subsequently formulate evidence - based strategies to enhance eHealth literacy, thereby fostering better health outcomes for this patient population.

Prior studies have established the importance of eHealth literacy in chronic disease management (10–12). However, limited research focuses on cardiovascular patients, particularly in China. Additionally, existing literature lacks consensus on the key sociodemographic predictors of eHealth literacy in this population. Therefore, this study addresses the following research questions: (1) What is the current level of eHealth literacy among cardiovascular disease patients? (2) Which sociodemographic and clinical factors significantly influence eHealth literacy levels?

The aim of this study is to assess eHealth literacy levels among cardiovascular disease patients and identify key determinants, providing evidence for targeted health communication strategies.

## 2 Materials and methods

### 2.1 Research subjects

We employed purposive sampling to recruit study participants. Cardiovascular disease patients who visited the cardiology department of a tertiary hospital in Shandong Province between January 2023 and August 2023 were selected. The inclusion criteria were as follows: (1) aged 18 years or older; (2) with a confirmed diagnosis of cardiovascular disease, including hypertension (ICD-10: I10-I15), coronary artery disease (I20-I25), heart failure (I50), and arrhythmias (I44-I49), as documented in their medical records. (3) having clear consciousness and the capacity to accurately comprehend the questionnaire content; and (4) providing voluntary consent to participate in the survey.

### 2.2 Methods

A purposive sampling strategy was employed between October 2023 and June 2024 at a tertiary hospital in Shandong Province. Eligible patients were approached during routine outpatient visits or post-hospitalization follow-ups. Research assistants explained the study purpose and obtained written informed consent.

A total of 720 questionnaires were distributed to cardiovascular patients, and 616 valid responses were retrieved, yielding an effective response rate of 85.5%. To minimize selection bias, recruitment was conducted across both urban and rural clinics, and all eligible individuals within the study period were invited unless they declined participation. Questionnaires were deemed invalid and excluded if they met any of the following criteria: (1) Missing values in ≥20% of eHEALS items; (2) Implausible responses (e.g., identical answers for all Likert-scale questions); (3) Completion time <5 min (determined via pilot testing as insufficient for thoughtful responses). Among 720 distributed questionnaires, 104 were excluded based on these criteria, yielding 616 valid responses for analysis.

A self-developed eHealth literacy survey questionnaire for cardiovascular disease patients was utilized. This questionnaire is composed of two principal components. The first part is designed to gather general information, encompassing patients’ age, gender, place of residence, educational attainment, marital status, and other fundamental demographic details. The second part is written by scholars including Norman, C. D (5), titled The eHealth Literacy Scale (eHEALS), which assesses competencies in searching, evaluating, and applying online health information. This scale predominantly comprises 8 items, each employing a five-point Likert scale. The response options are “strongly agree,” “agree,” “neutral,” “disagree,” and “strongly disagree”, which are assigned scores of 5, 4, 3, 2, and 1 point, respectively. Higher aggregate scores signify greater eHealth literacy levels. A score of 24 or above is defined as the threshold for adequate eHealth literacy, enabling the categorization of patients into groups with adequate and inadequate eHealth literacy (13).

The translated eHEALS has been rigorously validated, demonstrating good reliability and validity (14). In this study, the questionnaire exhibited a Cronbach’s *α* coefficient of 0.895. All surveyors received standardized training from the research team. They meticulously explained the study’s objectives, significance, and completion guidelines to the patients. The survey was administered via an online questionnaire platform. After securing patients’ informed consent, they were directed to scan a QR code to complete the data collection online, ensuring a seamless and efficient data - gathering process.

### 2.3 Statistical analyses

All statistical analyses were carried out using SPSS software, version 26.0. For measurement data, descriptive statistics were presented as the mean ± standard deviation. The *t*-test was employed to assess differences between groups. Categorical variables were expressed as percentages, and group differences were evaluated using the chi - square test. To establish statistical significance, logistic regression analysis was performed, with statistical significance set at a threshold of *p* < 0.05.

## 3 Results

### 3.1 Results of general information

A total of 720 questionnaires were disseminated. Following data screening and the exclusion of invalid data, 616 valid questionnaires were retained, resulting in an effective recovery rate of 85.5%. Among the 616 patients with cardiovascular disease, 274 (44.5%) were aged over 50 years. The sample consisted of 381 males (61.9%) and 235 females (38.1%). A total of 315 patients (51.1%) had a high school education or lower. The majority of patients (272, 44.2%) resided in urban areas. The monthly income of most patients fell within the range of 2000–5,000 yuan (243, 39.4%). The distance from their residences to the medical institutions they frequently visited was mainly 2–5 kilometers (205, 33.3%). Further details of the general data are presented in Table 1.

**Table 1:** eHealth literacy scores of cardiovascular disease patients with different characteristics.

Variable	Group	Number of cases	Electronic literacy score		*χ^2^*	*p*
			Not qualified	Qualified		
Age	18–30 years	69 (11.2)	49 (13)	20 (8.4)	7.759	0.051
	31–40 years	132 (21.4)	83 (22)	49(20.6)		
	41–50 years	141 (22.9)	74 (19.6)	67 (28.2)		
	>50 years	274 (44.5)	172 (45.5)	102 (42.9)		
Gender	Male	381 (61.9)	227 (60.1)	154 (64.7)	1.34	0.268
	Female	235 (38.1)	151 (39.9)	84 (35.3)		
Education level	High school and below	315 (51.1)	202 (53.4)	113 (47.5)	25.072	<0.001
	Associate degree	128 (20.8)	94 (24.9)	34 (14.3)		
	Bachelor’s degree	148 (24.0)	67 (17.7)	81 (34.0)		
	Master’s and above	25 (4.1)	15 (4.0)	10 (4.2)		
Residence	Urban	272 (44.2)	170 (45)	100 (42.9)	0.661	0.719
	Town	180 (29.2)	106 (28)	74 (31.1)		
	Rural or Suburban	164 (26.6)	102 (27)	62 (26.1)		
Occupation	Agricultural workers	151 (24.5)	80 (21.2)	71 (29.8)	17.84	0.058
	Technical professionals	99 (16.1)	53 (14.0)	46 (19.3)		
	Service personnel	29 (4.7)	18 (4.8)	11 (4.6)		
	Freelancers	23 (3.7)	18 (4.8)	5 (2.1)		
	Workers	37 (6.0)	26 (6.9)	11 (4.6)		
	Company employees	96 (15.6)	61 (16.1)	35 (14.7)		
	Government employees, civil servants, etc.	38 (6.2)	24 (6.3)	14 (5.9)		
	Housewives/Househusbands	13(2.1)	9(2.4)	4(1.7)		
	Unemployed	23 (3.7)	15 (4.0)	8 (3.4)		
	Retirees	57 (9.3)	44 (11.6)	13 (5.5)		
	Others	50 (8.1)	30 (7.9)	20 (8.4)		
Marital status	Married	308 (81.5)	203 (85.3)	511 (83.0)	4.904	0.179
	Single	51 (13.5)	31 (13.0)	82 (13.3)		
	Divorced	8(2.1)	1(0.4)	9(1.5)		
	Widowed	11(2.9)	3(1.3)	14(2.3)		
Monthly income	<2000 yuan	132 (21.4)	77 (20.4)	55 (23.1)	1.478	0.687
	2000–5,000 yuan	243 (39.4)	154 (40.7)	89 (37.4)		
	5,000–10,000 yuan	189 (30.7)	113 (29.9)	76 (31.9)		
	>10,000 yuan	52 (8.4)	34 (9.0)	18 (7.6)		
Sleep quality	Very good	111 (18.0)	52 (13.8)	59 (24.8)	20.5	<0.001
	Good	200 (32.5)	114 (30.2)	86 (36.1)		
	Average	247 (40.1)	171 (45.2)	76 (31.9)		
	Poor	52 (8.4)	36 (9.5)	16 (6.7)		
	Very poor	6 (1.0)	5 (1.3)	1 (0.4)		
Distance to medical institution	<1 km	101 (16.4)	41 (10.8)	60 (25.2)	24.692	<0.001
	1–2 km	125 (20.3)	74 (16.9)	51 (21.4)		
	2–5 km	205 (33.3)	138 (36.5)	67 (28.2)		
	>5 km	185 (30)	125 (33.1)	60 (25.2)		
Use of medical information websites or search engines	Yes	277 (45)	148 (39.2)	129 (54.2)	13.364	<0.001
	No	339 (55)	230 (60.8)	109 (45.8)		
Attention to own health status	Often concerned	301 (48.9)	208 (55)	93 (39.1)	15.638	<0.001
	Occasionally concerned	281 (45.6)	154 (40.7)	127 (53.4)		
	Never concerned	34 (5.5)	16 (4.2)	18 (7.6)		
Satisfaction with current health information sources	Very satisfied	329 (53.4)	167 (44.2)	162 (68.1)	39.502	<0.001
	Satisfied	111 (18.0)	87 (23.0)	24 (10.1)		
	Neutral	103 (16.7)	71 (18.8)	32 (13.4)		
	Dissatisfied	44 (7.1)	36 (9.5)	8 (3.4)		
	Very dissatisfied	29 (4.7)	17 (4.5)	12 (5.0)		
Proactive Health Awareness	Very knowledgeable	99 (16.1)	71 (18.8)	28 (11.8)	32.248	<0.001
	Generally knowledgeable	191 (31.0)	141 (37.3)	50 (21.0)		
	Only heard of	98 (15.9)	52 (13.8)	46 (19.3)		
	Never heard of	228 (37.0)	114 (30.2)	114 (47.9)		
Participation in Health Education Programs	Yes	230 (37.3)	173 (45.8)	57 (23.9)	39.879	<0.001
	No	226 (36.7)	105 (27.8)	121 (50.8)		
	Not aware	160 (26.0)	100 (26.5)	60 (25.2)		
Smoking status	Frequently smoke	88 (14.3)	54 (14.3)	34 (14.3)	0.359	0.949
	Occasionally smoke	44 (7.1)	27 (7.1)	17 (7.1)		
	Quit smoking	140 (22.7)	83 (22.0)	57 (23.9)		
	Never smoked	344 (55.8)	214 (56.6)	130 (54.6)		
Drinking status	Never drink	404 (65.6)	240 (63.5)	164 (68.9)	3.109	0.375
	1–2 times per week	158 (25.6)	101 (26.7)	57 (23.9)		
	At least 3 times per week	33 (5.4)	21 (5.6)	12 (5.0)		
	Almost every day	21 (3.4)	16 (4.2)	5 (2.1)		
Weekly exercise	Never exercise	248 (40.3)	150 (39.7)	98 (41.2)	4.382	0.223
	1–2 times per week	212 (34.4)	125 (33.1)	87 (36.6)		
	3–5 times per week	108 (17.5)	67 (17.7)	41 (17.2)		
	>5 times per week	48 (7.8)	36 (9.5)	12 (5.0)		

### 3.2 eHealth literacy scores among cardiovascular disease patients

The eHealth literacy scores of cardiovascular disease patients averaged 20.46 ± 9.54, with a passing rate of 38.6%. The mean score across all items was 2.55 ± 1.19. Specifically, for the items related to the capability of accessing and applying internet health information, evaluating internet health information and services, and making decisions based on such information and services, the mean scores were 2.49 ± 1.18, 2.67 ± 1.32, and 2.66 ± 1.35, respectively. Among individual items, the statement “I possess the necessary skills to evaluate whether the information found on the internet is useful” received the highest score, while “I know what kind of health information can be found on the internet” had the lowest score. Further details are presented in Table 2.

**Table 2:** eHealth literacy scores of cardiovascular disease patients.

Dimension	Item content	Score
Internet health information services and application capabilities		2.49 ± 1.18
	I know what kind of health information can be found on the internet	2.03 ± 1.11
	I know where to find useful health information online	2.65 ± 1.43
	I know how to find useful information on the internet	2.59 ± 1.40
	I know how to use the internet to search for answers to my health questions	2.60 ± 1.37
	I know how to use the health information I find on the internet to solve problems	2.59 ± 1.37
Evaluation capability of internet health information and services		2.67 ± 1.32
	I possess the necessary skills to evaluate whether the information found on the internet is useful	2.68 ± 1.37
	I can distinguish the quality of health information on the internet	2.67 ± 1.37
Decision-making capability of internet health information and services		2.66 ± 1.35
	I can confidently use the information found on the internet to make health-related decisions	2.66 ± 1.35
Total mean score		2.55 ± 1.19
Total score		20.46 ± 9.54

### 3.3 Binary logistic analysis of factors influencing eHealth literacy in cardiovascular disease

We employed eHealth literacy qualification as the dependent variable (coded as: unqualified = 0, qualified = 1). Binary logistic regression analysis was then performed, with the factors identified as significant in the univariate analysis serving as independent variables. The detailed coding of these variables is provided in Table 3.

**Table 3:** Variable coding.

Variable	Coding
Age	18–30 years = 1,31–40 years = 2,41–50 years = 3,>50 years = 4
Education level	High school and below = 1, Associate degree = 2, Bachelor’s degree = 3, Master’s degree and above = 4
Sleep quality	Very poor = 1, Poor = 2, Average = 3, Good = 4, Very good = 5
Distance to medical institution	Less than 1 kilometer = 1, 1–2 kilometers = 2, 2–5 kilometers = 3, More than 5 kilometers = 4
Use of medical information websites or search engines	Yes = 1, No = 0
Attention to own health status	Never concerned = 1, Occasionally concerned = 2, Often concerned = 3
Satisfaction with current health information sources	Very dissatisfied = 1, Dissatisfied = 2, Neutral = 3, Satisfied = 4, Very satisfied = 5
Proactive health awareness	Never heard of it = 1, Only heard of it = 2, Some understanding = 3, Fully understood = 4
Participation in health education programs	Yes = 1, No = 0

Pearson correlation analysis was conducted to assess multicollinearity. The results revealed that all correlation coefficients between variables were below 0.8, indicating no high linear associations among the variables, thus allowing for further analysis. The results of the Pearson correlation analysis are presented in Table 4.The findings of the binary logistic analysis revealed that several factors were associated with eHealth literacy qualification among cardiovascular disease patients. Specifically, education level, sleep quality, residing in close proximity to a medical institution (distance < 5 km), prior utilization of medical information websites or search engines, as well as the interaction between proactive health awareness and utilization of medical information websites or search engines were all important determinants (*p* < 0.05).The results of this analysis are presented in Table 5.

**Table 4:** Pearson correlation coefficients between variables.

		Education level	Age	Use of medical information websites or search engines	Attitude toward health online information	Satisfaction with current health information sources	Attention to own health status	Sleep quality	Distance to medical institution	Participation in health education programs
Education level	Pearson correlation	1	−0.476^**^	0.110^**^	−0.095^*^	0.022	−0.025	−0.152^**^	−0.124^**^	−0.018
	*P*		0.000	0.006	0.018	0.579	0.531	0.000	0.002	0.661
Age	Pearson correlation	−0.476^**^	1	−0.052	0.156^**^	−0.063	0.121^**^	0.111^**^	0.029	0.079
	*P*	0.000		0.197	0.000	0.116	0.003	0.006	0.469	0.050
Use of medical information websites or search engines	Pearson correlation	0.110^**^	−0.052	1	0.080^*^	−0.171^**^	−0.022	−0.072	−0.078	−0.036
	*P*	0.006	0.197		0.048	0.000	0.594	0.076	0.053	0.378
Attitude toward health online information	Pearson correlation	−0.095^*^	0.156^**^	0.080^*^	1	−0.090^*^	0.358^**^	−0.002	0.003	0.304^**^
	*P*	0.018	0.000	0.048		0.026	0.000	0.951	0.949	0.000
Satisfaction with current health information sources	Pearson correlation	0.022	−0.063	−0.171^**^	−0.090^*^	1	−0.012	0.140^**^	0.002	−0.001
	*P*	0.579	0.116	0.000	0.026		0.771	0.000	0.968	0.975
Attention to own health status	Pearson correlation	−0.025	0.121^**^	−0.022	0.358^**^	−0.012	1	0.134^**^	0.081^*^	0.302^**^
	*P*	0.531	0.003	0.594	0.000	0.771		0.001	0.043	0.000
Sleep quality	Pearson correlation	−0.152^**^	0.111^**^	−0.072	−0.002	0.140^**^	0.134^**^	1	0.084^*^	0.047
	*P*	0.000	0.006	0.076	0.951	0.000	0.001		0.038	0.241
Distance to medical institution	Pearson correlation	−0.124^**^	0.029	−0.078	0.003	0.002	0.081^*^	0.084^*^	1	−0.026
	*P*	0.002	0.469	0.053	0.949	0.968	0.043	0.038		0.520
	*P*	0.166	0.858	0.000	0.000	0.000	0.000	0.025	0.243	0.129
Participation in health education programs	Pearson correlation	−0.018	0.079	−0.036	0.304^**^	−0.001	0.302^**^	0.047	−0.026	1
	*P*	0.661	0.050	0.378	0.000	0.975	0.000	0.241	0.520	

“*” stands for *P*<0.05; “**” stands for *P*<0.01.

**Table 5:** Logistic regression analysis of eHealth literacy in cardiovascular disease.

Variable	*β*	*SE*	*Wald χ^2^*	*p*	OR	95% CI Upper Limit	95% CI Lower Limit
Education level			5.422	0.143			
Associate degree	1.441	1.376	1.097	0.295	4.226	0.285	62.743
Bachelor’s degree	2.723	1.327	4.211	0.040	15.219	1.130	204.998
Master’s and above	1.833	1.508	1.476	0.224	6.251	0.325	120.227
Sleep quality			14.304	0.006			
Poor	−0.628	0.306	4.205	0.040	0.534	0.293	0.973
Average	−1.064	0.295	13.027	0.000	0.345	0.194	0.615
Good	−0.784	0.446	3.091	0.079	0.457	0.191	1.094
Very good	−1.999	1.246	2.574	0.109	0.135	0.012	1.557
Distance to medical institution			12.479	0.006			
1–2 km	−0.945	0.339	7.752	0.005	0.389	0.200	0.756
2–5 km	−0.876	0.302	8.439	0.004	0.416	0.230	0.752
>5 km	−0.991	0.309	10.270	0.001	0.371	0.203	0.681
Use of medical information websites or search engines	1.189	0.571	4.337	0.037	3.284	1.072	10.055
Attention to Own Health Status			0.665	0.717			
Occasionally	0.028	0.252	0.012	0.912	1.028	0.627	1.686
Often concerned	0.388	0.489	0.629	0.428	1.474	0.565	3.846
Satisfaction with current health information sources			6.787	0.148			
Dissatisfied	−0.229	0.345	0.441	0.507	0.795	0.404	1.565
Neutral	0.081	0.349	0.054	0.816	1.085	0.547	2.150
Satisfied	−0.360	0.491	0.538	0.463	0.698	0.267	1.825
Very satisfied	1.083	0.522	4.299	0.038	2.954	1.061	8.222
Proactive health awareness			5.895	0.117			
Only heard of it	0.482	0.481	1.001	0.317	1.619	0.630	4.158
Some understanding	0.515	0.537	0.918	0.338	1.673	0.584	4.797
Fully understood	1.077	0.484	4.956	0.026	2.935	1.137	7.573
Participation in health education programs			5.306	0.070			
Yes	0.391	0.264	2.191	0.139	1.478	0.881	2.479
Unclear	−0.554	0.401	1.910	0.167	0.575	0.262	1.261
Proactive health awareness * use of medical information websites or search engines			10.779	0.013			
Only heard of *Have used medical information websites or search engines	−1.073	0.680	2.492	0.114	0.342	0.090	1.296
Some understanding* Have used medical information websites or search engines	−0.589	0.778	0.573	0.449	0.555	0.121	2.549
Fully understood* Have used medical information websites or search engines	−1.979	0.678	8.527	0.003	0.138	0.037	0.522
Age			4.200	0.241			
31–40 years	1.928	1.292	2.226	0.136	6.877	0.546	86.600
41–50 years	2.376	1.259	3.561	0.059	10.765	0.912	127.030
>50 years	2.329	1.235	3.557	0.059	10.267	0.913	115.503
Age * education level			6.125	0.727			
31–40 years*Associate degree	−2.217	1.581	1.967	0.161	0.109	0.005	2.414
31–40 years*Bachelor’s degree	−1.549	1.416	1.197	0.274	0.212	0.013	3.407
31–40 years *Master’s and above	0.623	2.048	0.093	0.761	1.865	0.034	103.303
41–50 years*Associate degree	−1.128	1.478	0.583	0.445	0.324	0.018	5.866
41-50 years*Bachelor’s degree	−1.775	1.443	1.515	0.218	0.169	0.010	2.863
41-50 years*Master’s and above	−1.575	1.833	0.739	0.390	0.207	0.006	7.514
>50 years* Associate degree	−1.781	1.472	1.463	0.226	0.169	0.009	3.017
>50 years* Bachelor’s degree	−1.604	1.422	1.272	0.259	0.201	0.012	3.267
>50 years*Master’s and above	−1.902	1.996	0.907	0.341	0.149	0.003	7.473

“*“*” is to represent a cross-analysis between two variables.

## 4 Discussion

### 4.1 Current status of eHealth literacy among cardiovascular disease patients

The survey findings reveal that the eHealth literacy score of cardiovascular disease patients is 20.46 ± 9.54. This value is lower than that of the general health check-up population (15), hospitalized patients with chronic kidney disease (16), hospitalized respiratory disease patients (17), and cancer patients (13). Nevertheless, it is marginally higher than the eHealth literacy score of the older adult. These results imply that there exists substantial potential for improving the eHealth literacy levels among cardiovascular disease patients.

Previous research has demonstrated that, both globally and domestically, the eHealth literacy levels of chronic disease patients are generally suboptimal (18–21), aligning with the outcomes of our study. This underscores the pressing necessity to enhance eHealth literacy among chronic disease patients, particularly in the face of an aging demographic and the accelerating pace of digitalization.

Notably, the dimension of “internet health information services and application capabilities” received the lowest score. This suggests that cardiovascular disease patients lack clarity regarding the utilization of the internet for acquiring health knowledge and information.

The relatively low eHealth literacy among cardiovascular disease patients may be attributed to the fact that a substantial proportion of them are middle - aged and older adult individuals. These patients typically have less frequent exposure to electronic devices, which in turn restricts their capabilities in accessing, evaluating, and applying online health information (22).

Healthcare professionals play a pivotal role in addressing this issue. They can assist patients by disseminating reliable online health knowledge resources, fostering an environment that encourages patients to engage in active learning, and promoting shared decision - making processes. This approach enables patients to make well - informed health - related decisions.

Notwithstanding the extensive presence of online health information, its complexity and variable quality pose significant challenges. Even younger demographics may encounter difficulties in discerning the quality of such information. This complexity can impede patients from making accurate health decisions based on online resources (23).

Consequently, healthcare professionals are urged to offer comprehensive training in eHealth. This includes curating a list of reliable health information websites, designing professional information - sharing platforms tailored to patients’ needs, and recommending trustworthy eHealth products and wearable devices (24–26). Such initiatives would empower patients to access, comprehend, evaluate, and apply relevant health information more effectively, ultimately leading to an improvement in their overall health status.

### 4.2 Analysis of factors influencing eHealth literacy among cardiovascular disease patients

The survey findings reveal that education level, sleep quality, proximity to medical institutions, utilization of medical information websites or search engines are determinants of eHealth literacy attainment in cardiovascular disease patients.

Using high school education or below as the reference group, individuals with undergraduate-level educational attainment demonstrated 15.219 times higher odds of achieving adequate eHealth literacy (OR: 15.219, 95% CI: 1.130–204.998). This aligns with previous research showing education level strongly predicts digital health competency through enhanced information processing and critical thinking skills (27). Therefore, health education interventions should particularly target less-educated populations through: (1) Developing tiered education programs using visual aids and simplified language for low-literacy groups; (2) Implementing community-based digital navigation services to bridge the “last mile” of health information access; (3) Creating hospital-community linkage mechanisms for sustained health education support.

The e-health literacy among the general population with relatively poor sleep quality is 0.534 (95% CI: 0.293, 0.973) and 0.345 times (95% CI: 0.194, 0.615) for those with very poor sleep quality. This is consistent with the results of previous studies, person with higher eHealth literacy are more likely to seek health information online, which may trigger excess searches online (28). Excessive internet use directly reduces netizens’ sleep duration and quality, leading to poor sleep.

The distances from medical institutions to home of 1–2, 2–5, and over 5 kilometers are 0.756 times (95% CI: 0.389–0.200), 0.416 (95% CI: 0.230–0.752), and 0.371 times (95% CI: 0.203–0.681) respectively, compared to the group whose distance from home is less than 1 kilometer. And contrary to initial hypotheses, administrative geographic labels (urban/town/rural) showed no direct association with eHealth literacy (*p* = 0.719). This finding challenges the utility of broad rural–urban dichotomies in digital health research. Instead, functional access barriers—proximity to medical institutions and community health education availability—emerged as stronger predictors, suggesting that physical and informational accessibility, rather than residency classification, may better explain disparities.

Patients residing in closer proximity to medical institutions, benefit from better access to comprehensive medical services (29). This likely leads to a greater emphasis on personal health, contributing to a higher perceived level of health literacy. This observation implies that health and wellness authorities should optimize the distribution of medical resources to narrow the urban - rural divide and safeguard patients’ equitable access to healthcare services (30, 31). And policymakers should prioritize expanding telehealth infrastructure and community-based training in underserved areas, regardless of administrative boundaries.

Some patients with high active health and more engine use have a low level of e-health literacy, which may be related to distrust of content in the network. Most patients who have previously utilized medical search engines demonstrate proficiency in acquiring health information online. They are more likely to be skilled in exploring diverse information - gathering strategies (32). Those who used such websites or search engines were 3.284 times more likely to be e-health literacy eligible than those who did not. Moreover, they tend to be more satisfied with their current health information sources, which is associated with a higher perceived eHealth literacy. This highlights the necessity for eHealth knowledge dissemination initiatives, such as health knowledge seminars and online health education campaigns. These efforts should promote reliable sources of cardiovascular health information, including search engines and specialized information portals (33).

Previous studies have demonstrated an inverse correlation between age and eHealth literacy, with younger populations exhibiting superior capabilities in health information seeking, evaluation, and application due to earlier exposure and adaptability to digital tools (34). Furthermore, aging-related cognitive decline diminishes older adults’ capacity to process electronic health information, compounded by distinct health needs and insufficient adaptability to eHealth technologies, collectively contributing to lower eHealth literacy levels in older adult populations (35). Occupation has conventionally been identified as a determinant of eHealth literacy, with technology-intensive professionals (e.g., education, healthcare, IT) demonstrating enhanced literacy levels attributable to higher socioeconomic status and routine digital tool utilization (36).

Notwithstanding theoretical expectations regarding the substantial influence of age and occupation on eHealth literacy, our regression analysis revealed no statistically significant associations for either variable. This discrepancy may stem from age-related sampling bias, as 44.5% of participants exceeded 50 years old. The restricted variability in eHealth literacy within this subgroup, potentially attributable to comparable digital health technology exposure and skill levels, might have attenuated the independent age effect. Additionally, age might exert indirect effects on eHealth literacy through mediating variables such as educational attainment and prior utilization of medical search engines, thereby diminishing its direct explanatory power. The occupational variable’s significance could be obscured by interaction bias with age, given the potential overrepresentation of older participants in low-digital-literacy occupations (e.g., retirees, traditional industry workers), consequently weakening the overall occupational effect magnitude.

Although the eHEALS scale effectively measures general eHealth literacy, it does not assess patients’ ability to use emerging tools such as mobile health apps or wearable devices. Future interventions could combine targeted educational materials with innovative case management via remote monitoring technologies. For example, integrating wearable device data into patient portals may facilitate personalized feedback and improve health outcomes. Based on our findings, critical next steps include: (1) Developing pilot training programs to test the efficacy of curated eHealth resources; (2) Designing longitudinal studies to evaluate the impact of wearable device integration on disease management; (3) Collaborating with technology developers to create patient-centered eHealth tools with simplified interfaces.

This study has several limitations. First, the purposive sampling from a single tertiary hospital limits generalizability, as participants may represent a more medically engaged subgroup compared to the broader CVD population. Second, the online data collection methodology likely excluded individuals with lower digital literacy or limited internet access, potentially inflating eHealth literacy estimates. While this approach ensured standardized administration, it introduces selection bias that future studies could mitigate through mixed-methods designs combining online and community-based recruitment. Finally, this study is limited by the potential age distribution bias in the sample (44.5% aged over 50 years), which may obscure the genuine effects of age and occupational characteristics. Future research will employ stratified sampling to balance age and occupational distributions while expanding the young population sample to enhance statistical power. Additionally, the current investigation inadequately explores potential mediating pathways through which age influences eHealth literacy. Subsequent studies should quantify age-related indirect effects using structural equation modeling to elucidate comprehensive causal mechanisms.

## Data Availability

The datasets presented in this article are not readily available because the policies and confidentiality agreements adhered to in our laboratory, we regretfully cannot furnish the raw data. Requests to access the datasets should be directed to WW, 147078729@qq.com.
